# The Impact of Negative Energy Balance in Holstein-Friesian Cows on the Blood Concentrations of Interleukin-6 and Plasminogen

**DOI:** 10.3390/metabo14100548

**Published:** 2024-10-14

**Authors:** Kalina Wnorowska, Krzysztof Młynek, Kamila Puppel

**Affiliations:** 1 Institute of Zootechnics and Fisheries, University of Siedlce, B. Prusa 14, 08-110 Siedlce, Poland; kw88137@stud.uws.edu.pl; 2 Institute of Animal Sciences, Departments of Animal Breeding, Warsaw University of Life Sciences, Ciszewskiego 8, 02-786 Warsaw, Poland; kamila_puppel@sggw.edu.pl

**Keywords:** dairy cows, body condition, negative energy balance, lipolysis, pro-inflammatory proteins

## Abstract

**Background/Objectives:** The negative energy balance activaties of spontaneous lipolysis. This may promotes inflammation within the adipose tissue. The aim of the study was to explain the development of inflammation during increased lactogenesis. It was hypothesized that lipolysis contributes synthesis of interleukin-6 and plasminogen. **Methods:** The study was in production conditions carried out using Holstein-Friesian cows. The period studied covered time of early lactation. **Results:** Up to the peak of lactation, milk yield strongly influenced the rate of loss of body condition. This had an impact on with the intensity of the release of the fatty acids. In both cases this relationships strengthened to the peak of production. Oobserved tendencies towards a decrease in the concentration of glucose and an increase in that of leptin. Loss of the body condition and the release of NEFA were were influencing to affect the blood concentrations of interleukin-6 and plasminogen. We have shown that IL-6 has a relatively strong correlation with the NEFA. They correlate with IL-6 independently of EB influence. This may suggest independent associations between these variables, which could potentially be applied in practice. **Conclusions:** The NEFA release in the long term can increase the inflammatory response within adipose tissue and can intensify the release of interleukin-6 and plasminogen. It is likely that in the initial stage of lactogenesis, the inflammatory process developing within adipose tissue is physiologically justified. Our results can provide background to this little-described area of research.

## 1. Introduction

The transition period and increased lactogenesis after calving cause a cascade of changes in dairy cows aimed at correcting the negative balance of energy (NEB) used for production [1]. This initiates spontaneous lipolysis of stored fat reserves in the adipocytes, which increases catabolism of non-esterified fatty acids (NEFAs) [2]. However, lipolysis increased by the intensity of developing lactogenesis does not proceed in proportion to the capacity for oxidation of NEFAs in the liver. A surplus of these acids burdens liver function in terms of biotransformation of metabolites formed during this period [3]. The body’s physiological response to a systemic increase in NEFAs and β-hydroxybutyrate (BHBA) is to increase blood flow through the liver, which additionally overburdens it [1,4,5]. This increases the occurrence of metabolic disorders associated with production [6]. An adverse effect of catabolism of NEFAs in the liver is increased production of reactive oxygen species [7]. Their presence in turn is conducive to the generation of oxidative stress, which can impair immunosuppression in cows [8,9,10]. The consequences of NEB not only can adversely affect the health of cows, but are also a cofactor in the deterioration of production indicators in dairy cow herds.

One of the lesser known consequences of NEB in cattle is its contribution to the occurrence of inflammation in the structures of adipose tissue (AT), induced by spontaneous lipolysis. The catabolism occurring in adipocytes also initiates the remodeling of adipose tissue [11]. This state begins to increase the formation of pro-inflammatory proteins, including cytokines (IL-6) and plasminogen (PL) [12]. It is likely that NEB can indirectly contribute to synthesis of plasminogen (PL), as this pro-inflammatory protein is produced in the liver parenchyma and in the adipocytes, sites which are closely associated with changes caused by NEB [13,14]. The cytokines, particularly those secreted from adipose, liver, co-regulate lipids and glucose metabolism related to lactation. Quite important is the fact that feed intake and nutrient utilization in ruminants may be strictly controlled by several different types of cytokines. IL-6 is involved in adipose tissue oxidation, insulin sensitivity, and anti-inflammatory processes [15,16]. Plasminogen is an abundant plasma protein that exists in various forms, acting as a cofactor influencing immune and inflammatory processes [17,18]. One of the functions of plasminogen is the so-called complement system and plays a key role in resolving inflammation [19,20]. The spontaneous mobilization of free fatty acids and decreased glucose storage observed in human adipose tissue results, among others, in the production of plasminogen activator-1, the main physiological inhibitor of plasminogen [21,22].

Research by Contreras et al. (2018) [23] showed that pro-inflammatory cytokines, such as IL-6, are produced mainly in immune cells located in the adipocytes. Therefore, an increase in the content of IL-6 in the blood may indicate an intensification of changes associated with metabolism of lipoproteins and oxidation of fatty acids. This is supported by results obtained by Wankhade et al. (2019) [16] in a study elucidating the effect of lactation intensity and dry matter consumption on activation of the immune system in cows during the transition period. These findings are in agreement with those published by Karis et al. (2020) [24], who showed that IL-6 was associated with metabolic dysfunctions. They indicate that an energy imbalance in cows in the perinatal period may be an indirect cause of this situation. Moreover, Sabzikar et al. (2023) [25] found that the immunosuppressive effect of pregnancy is one of the causes of increased IL-6. As all of these changes are associated with intensive energy use, they initiate NEB, leading to the activation of lipolysis, which most often takes a spontaneous course at the onset of lactogenesis.

The mechanism of the plasminogen (PL) activator plays a key role in numerous pathological processes, including inflammation in the adipose tissue (AT) [26]. Activators of PL production in the body include acute phase proteins (inflammatory cytokines) and weakening of the action of insulin [27]. Yudkin et al. (1999) [28] showed that even 30% of cytokines produced in humans, including interleukin-2 and -6 (IL-2 and IL-6), may come from AT. This is confirmed by the research Zachut (2015) [29], which demonstrated increased inflammatory and acute phase protein concentrations in the adipose tissue of cows with higher rates of lipolysis. However, the connections between markers of NEB and the intensity of synthesis of pro-inflammatory proteins are still little understood. The concept of our research resulted from the need to analyze the consequences of NEB as a broader perspective for assessing the health of cows and intensification of milk production. The aim of the study was to explain the intensity of development of inflammation during increased lactogenesis. It was hypothesized that increased intensity of lactogenesis, which intensifies lipolysis, initiates synthesis of interleukin-6 and plasminogen, increasing their systemic concentrations.

## 2. Materials and Methods

### 2.1. Housing Conditions and Health of Cows

The material (blood) and necessary data for the study were collected in three experiments (commercial farms F1, F2, and F3). Holstein-Friesian (HF) cows were used in the study. The number of animals used on each farm was 15, 18, and 22, accounting for 43% to 49% of the respective herds. The cows were part of a separate feeding group, and their average lactation yield (±SD) was 9490 ± 303 kg (F1), 8375 ± 359 kg (F2), and 9544 ± 327 kg (F3). The cows’ average lactation number was 3.2 (F1), 3.1 (F2), and 2.9 (F3). Daily milk production (DMP) was used for the analysis. The production level was determined from measurements made in four stages of lactation (SL1–4). Measurements of daily milk production (DMP) were made according to the procedure described in the subsection ‘Collection of material for analysis’. The animals were kept in cowsheds with resting boxes and had free access to feed (a feed table) and water (automatic drinkers). Fans were used to keep the cows comfortable during hot weather. The cowsheds were in compliance with animal welfare standards, and the herds were under regular veterinary supervision.

### 2.2. Control of the Diet and Body Conditions of Cows

On all farms the cows’ diet was established on the basis of analysis of the share of nutrients making up the TMR (total mixed ration) [30]. The demand for nutrients was calculated for a body weight of 600 kg and 35 kg daily milk production, with 3.3% protein and 4.1% fat (INRAtion 4.06: INRA, France) [31]. The balance of the diet and the nutritional value of TMR in the groups are presented in Table 1.

The same diet ingredients were used in all groups. The levels of each component (kg/day/cow) were as follows: maize silage (22.7–25.5), barley (0.4–0.7), oat (0.5–0.9), wheat (0.5–0.7), triticale (0.9–1.4), rapeseed meal (0.7–1.2), soybean meal (2.1–2.4), NaCl (0.02), and chalk (0.1–0.2). The cows had unlimited access to salt licks, which provided supplementary microelements (NaCl—94.0%, Mg—0.20%, Co—0.18%, Zn—0.80%, Mn—0.83%, I—0.1%, Se—0.1%, compounds insoluble in water—4.0%).

The diet was adjusted on the basis of body condition score (BCS) and the amount of uneaten feed. The daily amount of uneaten feed was monitored about every three days. This was the basis for calculating dry matter intake (DMI).

Feed (TMR) was prepared in a feed wagon and supplied to the cows three times a day (on average every 8 h). Feed pushing was used to increase the accessibility of the feed. About two weeks before calving, the cows received a preparatory diet.

The energy balance (EB) was calculated from the energy supplied with the diet and its expenditure for milk production. Milk yield was corrected for energy, calculated from the content of fat, protein, and lactose according to Sjaunja et al. (1990) [32]. EB was calculated as daily energy intake (E_intake_)—energy used to produce milk and maintain lactogenesis (E_milk_). E_milk_ was expressed in MJ, using a conversion factor of 4.19 J/cal [33]. E_intake_ was calculated using information on the average dry matter intake (DMI) during the periods analyzed. These values were multiplied by the energy supplied with one kilogram of TMR (Table 1). The net energy demand was calculated on the basis of metabolic body weight (BW) as BW0.75 × 0.08. E_milk_, in kilograms of milk production, was calculated using the following algorithm: (0.0929 × fat%) + (0.0588 × protein%) + (0.0395 × lactose%) [34]. Energy balance was expressed in MJ NEL per day (EBMJNEL/d).

The body condition of the cows (average BCS) was assessed on a 5-point scale, independently by two individuals [35]. Subsequent assessments were made on the same days that samples were taken for analysis. BCS% was calculated on the basis of changes in body condition (BCS) between stages of lactation, expressed as the percentage loss of BCS. The differences were calculated each time in relation to the previous score. Body condition was one of the indicators of the course of lipolysis.

### 2.3. Collection of Material for Analysis

Cows were included in the analysis following a three-week period after parturition (convalescence) if they showed no somatic signs of infection (mastitis, abscesses, and wounds on the skin and hooves, swollen joints, or respiratory infections and without somatization caused by perinatal diseases). Cows qualified for the experiment also had to meet the condition related to the manifestation of estrus after calving. Cows in which such changes were not observed by the 40th day after calving were automatically excluded from the study. These criteria were meant to minimize error associated with the occurrence of factors that could influence the inflammatory markers analyzed: interleukin-6 (IL-6) and plasminogen (PL). Taking these criteria into account, blood was collected during four stages of lactation: SL1—21–28 days of lactation (period after convalescence), SL2—42–49 days of lactation (increased development of lactogenesis), SL3—63–70 days of lactation (peak of milk production), and SL4—147–154 days of lactation (positive energy balance).

Milk for analysis was collected twice a day (morning and evening). Each time, 1 mL per liter of milk obtained during milking was collected for analysis (Tru-Test milk meter Datamars livestock; New Zealand). DMP was determined on the same days when blood was sampled.

Blood for analysis was collected from the abdominal vein, before morning feeding. The material was collected into single-use tubes (MedLab-Products, Pruszków, Poland). Blood for plasma analysis was placed in tubes with a clot activator. Tubes containing sodium fluoride were used for analysis of glucose content (GLU). The blood was gently mixed to avoid haemolysis and then placed in a portable refrigerator with an internal temperature of about +4.0 °C ± 1.0 (CDF18; DanLab, Białystok, Poland). In the case of GLU, the tubes were placed on ice to inhibit glucose metabolism. The blood was immediately transported to the laboratory, where the plasma was separated by centrifuging (MPW-352RH centrifuge; DanLab, Poland; 1500× *g* at 4 °C for 20 min) and stored at ±75 °C until analysis.

### 2.4. Analytical Procedures

The composition of the milk was determined using the Bentley Combi 150 analyzer (Bentley Instruments, Inc., Chaska, MN, USA). Udder health, as the occurrence of inflammation, was assessed by analyzing milk collected before regular milking.

The markers used to characterize the course of lipolysis were the blood levels of non-esterified fatty acids (NEFAs), β-hydroxybutyrate (BHBA), glucose (GLU), and leptin (LEP). The content of BHBA was determined using Randox commercial kits (Randox Laboratories Ltd., Crumlin, UK) and read on a UV–Vis spectrophotometer (Varian Inc., Palo Alto, CA, USA). LEP content was determined by ELISA, using kits containing bovine antibodies (EIAab, Wuhan, China). GLU was determined using a commercial Randox test (Randox Laboratories Ltd., Crumlin, UK) and a UV-Vis spectrophotometer (Varian Inc., Palo Alto, CA, USA). The level of NEFAs was determined on the basis of the content of the following acids in the plasma: C16:0, C18:0, C18:1-t9, and C18:2. The lipid fraction was extracted with a mixture of hexane and isopropanol [36]. Fatty acids (FAs) were determined by gas chromatography with mass detection (GCMS: Agilent Technologies Inc., Wilmington, DE, USA). FAs were separated following their conversion to methyl esters (FAME) in a heating block (Thermo Fisher Scientific, Waltham, MA, USA) at 70 °C ± 0.5 [37]. Separation was carried out on a 100 m × 0.250 mm column (HP-88; SN:UST458414H, Agilent Technologies Inc., USA). The temperature programme was as follows: injector 250 °C; furnace 95 °C (5 min), 120 °C (15 °C/min for 15 min), 210 °C (25 °C/min for 30 min), 250 (20 °C/min for 5 min). Carrier gas flow (H): 0.7 mL/min. Identification and percentages were based on retention times and standards (Supelco 37, No:47885-U; Sigma Aldrich, St. Louis, MO, USA) using Chemstation software (A09.03 Agilent Technologies Inc., Wilmington, DE, USA).

### 2.5. Statistical Analysis

Data management and statistical analysis were performed in Statistica 13.0 software (Stat Soft Inc., Tulsa, OK, USA). The results were checked for normal distribution using the Kolmogorov–Smirnov test. Because the cows were from different farms, an analysis was performed to test the influence of diet (F1–3), using k-means clustering. The model included the actual energy consumption and DMI in the groups. Analysis of variance, taking into account the stage of lactation (SL1–4) and the BCS% changes during these periods, was carried out using a linear model (GLM) with repeated measures. The results were presented as means (least squares method—LSM) and standard error of the mean (SEM). The significance of differences between means was estimated using Duncan’s test at *p* ≤ 0.05. Following analysis of the tendencies observed for changes in the content of NEB markers, curvilinear correlations were calculated for the period of negative EB (SL1–3) and the period of EB restoration (SL4). The independent variable was determined prior to approximation of the model. The power of the model was determined using SEM and R^2^. Correlations between selected parameters were estimated using polynomial regression (*p* ≤ 0.05).

## 3. Results

One of the main reasons for the appearance of negative energy balance (NEB) in dairy cows after calving is the intensive development of lactogenesis. Table 2 shows that milk production during the lactation period increased up to the peak of lactation (SL3) on average by 6.7 kg (*p* ≤ 0.05). The highest rate of changes in lactogenesis was noted between SL1 and 2, as DMP increased on average by 0.39 kg/day during this time. In the next interval, lactogenesis developed at a slower rate, on average 0.21 kg/day (SL3). In the period from SL3 to SL4, the intensity of lactogenesis (DMP) decreased, on average by 0.08 kg/day. In comparison to SL1 and SL4, in the periods of increasing milk production (FL3–4), we noted lower content of protein and fat in the milk, on average by 0.15% (*p* ≤ 0.05), and higher content of lactose, on average by 0.17% (*p* ≤ 0.05).

As expected, the intensity of development of lactogenesis (DMP) influenced the markers we used to characterize the consequences of NEB. In the periods of increased changes in the development of lactogenesis (SL1–3), the BHBA concentration increased from 0.360 to 0.426 mmol L^−1^ (*p* ≤ 0.05). Following the peak of lactation (SL3), however, its content decreased, on average by 0.456 mmol L^−1^ (*p* ≤ 0.05). We observed a different tendency for glucose (GLU) in the blood, as more rapid changes in the development of lactogenesis were accompanied by a decrease in its concentration. The greatest difference was noted between SL1 and SL2, when the GLU concentration decreased on average by 0.33 mmol L^−1^ (*p* ≤ 0.05). In the next period (up to SL3), the decrease in its concentration was smaller, amounting to 0.16 mmol L^−1^ on average (*p* ≤ 0.05). The restoration of the GLU concentration following the peak of lactation (SL4) took place at a similar level, on average 0.15 mmol L^−1^ (*p* ≤ 0.05).

An important regulator of lipid metabolism as well as a feeling of satiety is leptin (LEP), analyzed in our study. Its lowest concentration was noted in the first period (SL1). However, a pronounced association was observed between LEP and the pace of changes in the development of lactogenesis. Up to the peak of lactation (SL3), we noted an increase in the LEP concentration in the blood, ranging from 0.25 to 0.11 ng mL^−1^ (*p* ≤ 0.05), whereas a marked decrease in the LEP concentration, on average by 0.37 ng mL^−1^ (*p* ≤ 0.05), was shown following the peak of lactation (SL4).

In the period of developing lactogenesis, a key factor in maintaining energy homeostasis in the cow is the physiological efficiency of metabolism of surplus NEFAs. Table 3 presents changes in the values of selected markers of lipid metabolism. The data show that up to SL3 (peak of lactation) the rate of loss of body condition (BCS%) remained similar, as the differences noted between stages of lactation were similar, amounting to −5.5% on average (*p* ≤ 0.05). A greater difference, but indicative of the restoration of body condition in the cows, was shown after the peak of production. The BCS% values during this period were on average +9.2 (*p* ≤ 0.05), which indicates that NEB was no longer present and the process of restoration of body condition was underway.

Levels of NEFAs analyzed in the study (Table 3) confirmed that the changes in BCS were accompanied by changes associated with NEFA concentrations in the blood. An upward trend in their concentration was observed from SL1 to the peak of production (SL3). The greatest differences in their content were usually noted during the most intensive development of lactogenesis (Table 2), between SL1 and SL2. Differences in the concentrations of NEFAs during this period ranged from 0.04 mmol L^−1^ for C18:0 (*p* ≤ 0.05) to 3.22 mmol L^−1^ for C16:0 (*p* ≤ 0.05). In contrast, between SL3 and SL4 there was a downward trend in the NEFA concentrations in the blood. The differences ranged from 0.70 mmol L^−1^ for C16:0 (*p* ≤ 0.05) to 3.29 mmol L^−1^ for C18:0 (*p* ≤ 0.05).

Characterization of cows in terms of the development of lactogenesis (Table 3) showed a significant effect of the SL on the concentrations of markers of the development of inflammation in the body. In the case of interleukin-6 (IL-6), the greatest increase in its concentration in the blood was noted during the period of the greatest development of lactogenesis (SL1–2), when differences of 10.58 ng L^−1^ (*p* ≤ 0.05) were observed. In the next period, lasting up to the peak of lactation (SL4), IL-6 increased only by 6.54 ng L^−1^ (*p* ≤ 0.05). However, in the period when restoration of body condition was generally observed in the cows (SL3–4), the IL-6 concentration showed a decreasing tendency, as confirmed by a difference amounting to 2.69 ng L^−1^ (*p* ≤ 0.05).

In the case of the blood concentration of plasminogen (PL), the tendency was similar to the case of IL-6 (Table 3). The greatest increase in the PL concentration, on average 0.46 ng L^−1^ (*p* ≤ 0.05), was noted in the period from SL1 to SL2. Up to the peak of milk production (SL3), the values no longer increased to that extent, although the differences remained statistically significant (0.29 ng L^−1^; *p* ≤ 0.05). After this time, a decreasing tendency in the concentration of this peptide was shown, as indicated by a difference amounting to 0.26 ng L^−1^ (*p* ≤ 0.05).

The tendencies observed for the consequences of NEB are confirmed by the values for the correlation coefficient (r) presented in Table 4. They show that the changes in DMP were significantly associated with BCS%. Up to the peak of lactation (SL3), DMP was positively correlated with BCS% (*p* ≤ 0.05). In addition, this relationship became stronger up to the peak of lactation (SL3), taking on values from 0.447 to 0.742 (*p* ≤ 0.05). In SL4, however, the r value was negative and the lowest (−0.359; *p* ≤ 0.05).

The previously described influence of the intensity of DMP on the course of lipolysis was also found for the changes in the blood concentration of NEFAs and BHBA. The concentrations of these substances were relatively strongly correlated with the rate of loss of body condition (BCS%; Table 4). The positive correlations between them were strongest up to SL3. Negative r values were noted in SL4, in the period of BCS restoration. Unsurprisingly, BCS% was associated with the intensity of NEFA release during lipolysis and the increase in accumulation of BHBA in the body. For these markers, the correlation coefficients with BCS% ranged from 0.143 to 0.452 in SL1 (*p* ≤ 0.05) and from 0.504 to 0.704 in SL3 (*p* ≤ 0.05). The exception was the C18:0 x BSC% correlation, which remained positive up to SL3 but became weaker. In SL4, when lactogenesis was less intensive, the r values were negative and ranged from −0.211 to −0.412 (*p* ≤ 0.05).

Glucose (GLU) and leptin (LEP), which has an anorectic effect, were relatively strongly correlated with BCS% (Table 4). The r values indicate that up to SL3 a greater loss of BCS was accompanied by a lower GLU concentration in the blood and a higher concentration of LEP. The r values noted at this time also indicate that the strength of these relationships increased up to SL3. In the period of restoration of body condition (SL4), the r values indicate a positive relationship between BCS% and GLU and a negative relationship with LEP (*p* ≤ 0.05). The relationships between GLU and LEP observed up to SL3 indicate that cows with a higher concentration of LEP at the same time had a lower blood glucose concentration and a more rapid loss of body condition (BSC%). The negative correlation (0.486; *p* ≤ 0.05) noted between GLU and IL-6 and positive correlations for BCS% x IL-6 up to SL3 (0.276–0.467; *p* ≤ 0.05) indicate that both GLU availability and the rate of loss of body condition were associated with the formation of pro-inflammatory IL-6 in the body. It is also significant that positive and relatively strong correlations, ranging from 0.294 to 0.731 (*p* ≤ 0.05), were shown between the concentrations of NEFAs and IL-6. Similar tendencies for the r values in the SLs were also shown for the concentration of plasminogen (PL). They also confirm the results presented in Table 3. The results presented in Table 4 indicate that the PL concentration was correlated with the BCS% in the analyzed SLs, with positive values up to SL3 and negative values in SL4. The PL concentration was also shown to be positively correlated with the concentrations of the pro-inflammatory protein IL-6 (0.361; *p* ≤ 0.05) and LEP (0.264; *p* ≤ 0.05). At the same time, we showed fairly strong and negative correlations between the intensity of GLU synthesis and levels of IL-6 and PL (−0.486 and −0.619, *p* ≤ 0.05).

**Figure 1:**
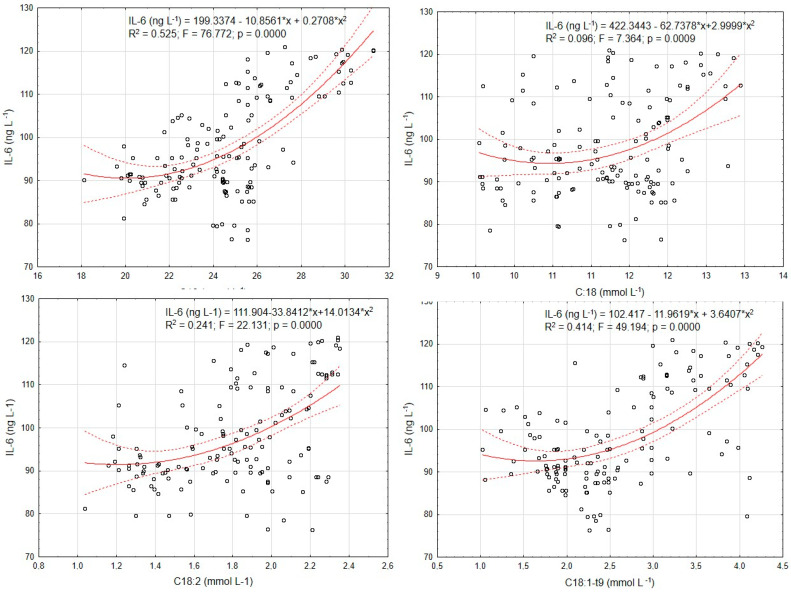
Regression coefficients lines for the dynamics of changes in interleukin-6 (IL-6) and lipolysis markers (dash line—confidence interval, solid line—power trendline).

In Figure 1, the results illustrating the dynamics of changes in IL-6 relative to the analyzed lipolysis markers, NEFAs, are presented. All analyzed regression models showed a positive slope. We demonstrated that the weakest marker was C18, where the applied regression model explained 9.6% of the variability in relation to IL-6. However, we also observed relatively strong associations with C16 and C18:1-t9. For these markers, the explained variability was approximately 52% and 41%, respectively (*p* = 0.000).

The data presented in Figure 1 suggest that IL-6 has a relatively strong correlation with the lipolysis parameters analyzed in our study. While Table 4 shows results indicating a relationship between EB and lipolysis markers (NEFAs) and IL-6, the data from Figure 1 indicate that NEFAs correlate with IL-6 independently of EB influence. This may suggest independent associations between these variables, which could potentially be applied in practice.

## 4. Discussion

Physiological changes taking place in dairy cows from calving to the start of restoration of body condition (BCS) following the peak of lactation influence many aspects of their health [4,5]. One of the commonly described effects is metabolic disorders associated with spontaneous lipolysis, which is initiated by intensive development of lactogenesis [2,6]. The studies cited showed that the spontaneity of the release of NEFAs became stronger as the peak of production approached. As expected, we observed a similar tendency in our study. The negative energy balance (NEB) developing together with lactogenesis increased the release of NEFAs. This is confirmed in studies by Drackley et al. (2001) [38] and Schoenberg and Overton (2011) [39], which showed that lipolysis can release about 3712 g NEFAs/day during NEB. In the first 30 days of lactation this can amount to even 30% of the total energy demand. The tendencies in the loss of BCS and concentrations of lipolysis markers noted in our study are consistent with these findings., although we noted less intensive release of NEFAs, and the rate of decline in BCS (amounting on average to 5%) was maintained up to about 67 days of lactation (peak of production). This seems significant because it indicates a longer period of production stress burdening the body, including the occurrence of inflammation within the adipose tissue. In early lactation, this is also important due to the excessive influx of NEFAs to the liver and unregulated hormonal balance. Studies by Bobe et al. (2004) [40] and Stefan et al. (2013) [41] indicate that the co-occurrence of these changes may increase the severity of metabolic dysfunctions. Our study additionally showed that excessive intensity of lipolysis can indirectly increase the production of pro-inflammatory proteins. The transition period is one of stressful times in the production of dairy cows. At this time, the metabolic stress is response to the hypermetabolic catabolic and the physiological homeostasis. In effect, cows are characterized by excessive lipomobilization and inflammatory dysfunctions [42].

Greenfield et al. (2000) [43] and Weber et al. (2013) [44] showed that the liver of cows in the transition period has a fairly efficient mechanism of adaptation to the increased demand for glucose. However, according to Aschenbach et al. (2010) [45], the availability of substrates is a factor that nevertheless limits its synthesis. This may be significant, especially given that up to the peak of lactation, reduced feed intake is usually observed in cows. This may be linked to the increased leptin concentration shown in our study during NEB. Aschenbach et al. (2010) [45] also draw attention to the metabolism of compounds such as lactates or amino acids, which also takes place in the liver. In the long term, this can affect liver function by impairing the functioning of hepatocytes, not only in terms of neutralization of NEFAs [38]. The mechanism of mitigation of pro-inflammatory responses induced during ATP production in the mitochondria of adipocytes (lipolysis) and liver cells (glycogenesis) can be impaired as well, Trayhurn (2013) [46]. We also observed the effect of impaired liver function in our study, as up to the peak of lactation (on average 67 days of lactation) the BHBA concentration increased while the glucose (GLU) concentration decreased. These tendencies were confirmed by the correlation coefficients obtained and are consistent with the direction of changes observed by Karis et al. (2020) [24]. In that study, between 21 days before calving and 21 days of lactation, cows with optimal BCS showed a marked increase in the concentrations of NEFAs by 0.51 mmol/L and of BHBA by 0.31 mmol/L, accompanied by a 1.521 mg/dL decrease in GLU. Significantly, that study showed a decrease in the insulin concentration at the same time, from 6.849 to 3.336 µg/dL. These results can to some extent be explained by the findings of Stefan et al. (2013) [41], who showed that GLU availability depends mainly on the metabolic efficiency of the liver. Thus, the sharp increase in the BHBA concentration can be linked to impairment of its function of lipid metabolism. The decreased blood concentration of GLU can be explained by decreased glycogenesis and limited availability of its main substrate, pyruvates absorbed in the rumen. During the period of increased lactogenesis, feed intake usually decreases. For this reason, up to the peak of lactation this factor largely determines NEB. An additional factor that may disturb the energy balance at this time is pathophysiological mechanisms on the glucose—fatty acid axis. De Koster and Opsomer (2013) [47] showed that a decrease in insulin sensitivity during the transition period may be physiologically justified. This mechanism seems to be an endogenous factor strengthening the transfer of energy and nutrients directly to mammary gland cells. However, the trends in GLU concentrations observed in our study indicate that reduced insulin sensitivity may persist longer. This can be concluded from the results of a study by Eckel et al. (2005) [48], who suggested that the main factor of this pathophysiology is a systemic excess of fatty acids. This hypothesis may explain why up to the peak of lactation we observed negative correlations between NEFAs and the GLU concentration and a positive r value for the rate of loss of body condition (BCS%).

When explaining the tendencies described for NEB markers, changes in the concentrations of leptin (LEP), which exerts an anorectic effect, should be considered as well, as greater production and a higher concentration of LEP may have reduced appetite more. This may have limited the consumption of nutrients for synthesis of GLU and indirectly impair its synthesis in the liver. This is indicated by the fairly high negative correlation of GLU with LEP, amounting to −0.589 (*p* ≤ 0.05). In contrast to our results, Çolakoğlu et al. (2017) [49] showed that the leptin concentration decreases during the development of NEB. These differences, however, can be explained by the period of lactation when the changes in LEP concentration were determined. In the study cited, this was the peripartum period, whereas we analyzed its concentration on average from 23 days of lactation.

The rate of changes in BCS during the period of intense development of lactogenesis generates another category of changes that can be associated with lipid metabolism during NEB. This concerns regulation of the secretion of inflammatory cytokines in the adipocytes, such as interleukin-6 (IL-6), associated with the response to the intensity and duration of lipolysis in the AT [23]. Up to the peak of production, lactation and pregnancy generate an enormous demand for energy and protein [50]. These processes intensify lipolysis, during which adipokines play an important regulatory role. As pro-inflammatory proteins, they are associated with pathophysiological changes taking place during NEB (spontaneous lipolysis, steatosis of the liver, or ketosis). Our study showed that until the NEB was resolved, the secretion of pro-inflammatory IL-6 was positively correlated with the rate of loss of BCS (BCS%) and the release of NEFAs. The tendencies observed in our study may be explained by the findings of Wankhade et al. (2019) [16] and Kabara et al. (2014) [51]. Wankhade et al. (2019) [16] showed that IL-6 is associated with the metabolism of fatty acid oxidation. This association was indirectly shown in our study, in which an increased BHBA concentration in the blood was accompanied by increased production of IL-6. Kabara et al. (2014) [51] showed that changes in the blood concentration of adiponectin secreted in fat cells in cows were negatively correlated with concentrations of NEFAs. In their opinion, a high systemic NEFA concentration during NEB is a factor predisposing cows to inflammatory disorders. The aetiology of this mechanism is determined by an increased risk of an uncontrolled inflammatory response of macrophages and monocytes during correction of the energy deficit from lipolysis Kabara et al. (2014) [51].

An interesting hypothesis was put forth by Wang and Ye (2015) [52], according to which it is the development of inflammation in the adipose tissue (AT) that initiates the mechanism of energy mobilization from the adipocytes, inhibits its storage, and indirectly even reduces feed intake. However, research by Ye and McGuinness (2013) [53] indicates that inflammation is most often due to hypoxia of AT. In that study, the authors showed that it was linked to stress responses in the adipocytes. In our opinion, this effect was enhanced, as the values of the correlation coefficient between the rate of loss of body condition (BCS%) and IL-6 and PL increased up to the peak of lactation. Ye and McGuinness (2013) [53] additionally showed that stress responses, which include a severe loss of BCS, can contribute to a reduction in adiponectin and generate pathophysiological changes, leptin expression, or even adipocyte death. This is consistent with results reported by Wallenius et al. (2002) [54] and with our own findings, as we noted positive correlations for the concentrations of LEP and IL-6 (r = 0.295; *p* ≤ 0.05). Kabara et al. (2014) [51] showed that excessive release of NEFAs and intensive glucose synthesis can induce oxidative stress. This mechanism was also explained by the intensive production of ATP in the mitochondria of cells involved in metabolism of NEFAs and GLU. We observed a similar consequence in our study, but in cows during NEB, which persisted up to the peak of lactation. At this time the more intensive release of NEFAs was accompanied by increased synthesis of IL-6 and PL. The strongest correlations were shown for C16:0 and C18:2 (r = 0.518 and 0.731; *p* ≤ 0.05). Our results are also in agreement with the findings of Kabara et al. (2014) [51] regarding the effects of GLU. In our study, increased GLU synthesis was accompanied by higher concentrations of IL-6 and PL (r = 0.846 and 0.619; *p* ≤ 0.05). This indicates that up to the resolution of NEB, the rate of loss of BCS/lipolysis is a factor increasing the risk of inflammation in the AT. Like Ye and McGuinness (2013) [53], Trayhurn (2013) [46] also explain the appearance of inflammation in the AT as a response to AT hypoxia as the release of NEFAs increases during lipolysis. The increase in the concentrations of IL-6 as daily milk yield/energy demand increased in our study can be explained by the findings of Contreras et al. (2018) [23] and Wankhade et al. (2019) [16], who showed that pro-inflammatory cytokines such as interleukin IL-6 are produced by adipocytes and immune cells residing in adipose tissue. Therefore more intensive lipolysis may be conducive to IL-6 production. In addition, Wankhade et al. (2019) [16] showed that IL-6 plays a fundamental role in the metabolism of lipoproteins and oxidation of fatty acids. This in turn explains the positive correlations obtained in our study for IL-6 and the rate of release of NEFAs, as well as the generation of BHBA in the liver owing to their surplus, although the BHBA concentration did not exceed the limits suggesting a risk of ketosis.

Our results are only to a small extent in agreement with the findings of Sabzikar et al. (2023) [25]. The cows used in that study had a similar IL-6 concentration to the animals in our study in the third week of lactation (98.93 ng L^−1^). However, up to that time, Sabzikar et al. (2023) [25] recorded higher levels of IL-6. This indicates a reduction in its concentrations over the course of development of lactation, whereas the reverse trend was observed in our study. This discrepancy may be due to the period when the authors tested the cows. In our opinion, the true picture of the IL-6 concentrations in that study may have been masked by the inflammatory response of the reproductive system, which was regenerating after calving. For this reason, the authors did not observe a reduction in the IL-6 concentration until the third week of lactation. We believe that the IL-6 concentration obtained in our study at that time (94.46 ng L^−1^) indicates an early stage of development of inflammation induced by lipolysis.

The knowledge gained in our studies indicates that a significant cofactor that can deepen the occurrence of inflammation in the body is spontaneous lipolysis. This is indicated by the correlations we found between the formation of pro-inflammatory protein content and lipolysis markers. In our opinion, the fact that these relationships were found in early lactation may have significant application potential, especially in the area of rationalization of production intensification in dairy herds. It also indicates the need to conduct research on traits that facilitate metabolic coping of cows with production stress. Understanding the physiological commitment of cytokines in ruminants may influence the improvement dairy cows health and the milk quality.

## Figures and Tables

**Table 1:** Nutritional value and nutrient balance of the diet (farms 1–3) during the analyzed lactation period in Holstein-Friesian cows (HF).

Parameter	F1	F2	F3
Number of cows	15	18	22
Dry matter	43.1	42.2	42.9
Protein	16.4	16.9	17.1
Fibre	19.2	18.9	19.4
Fat	2.5	2.4	2.6
Ash	8.0	7.8	8.3
Starch	22.7	23.1	22.8
Acid detergent fibre—ADF (%)	22.8	22.4	22.6
Neutral detergent fibre—NDF (%)	39.5	38.6	38.9
Physically effective NDF—peNDF (%)	30.6	30.8	31.1
UFL	21.5	20.5	21.3
PDIN (g)	2459	2318	2473
PDIE (g)	2201	2197	2185
Energy (MJ NEL):			
Requirement	151.7	145.3	149.8
Intake	153.2	147.4	151.6
Balance	+1.5	+2.1	+1.8
Dry matter intake—DMI (kg/day)	23.1	24.4	23.5

**Table 2:** Milk production and composition in stages of lactation (SL) and markers of changes caused by NEB.

Trait	Stage of Lactation (SL)				SEM	Correlation DL x
	SL1	SL2	SL3	SL4		
Days of lactation (DL)	22.6 ^d^	45.4 ^c^	67.2 ^b^	152.9 ^a^	9.2	**−**
EB (MJNEL/d)	−7.7 ^c^	−11.3 ^b^	−13.6 ^a^	2.3 ^d^	3.8	0.658 *
DMP (kg)	23.5 ^d^	32.4 ^b^	36.9 ^a^	29.8 ^c^	0.6	−0.467 *
Contents in milk (%):						
Protein	3.42 ^a^	3.28 ^b^	3.27 ^b^	3.44 ^a^	0.22	−0.438 *
Fat	4.31 ^a^	4.12 ^c^	4.14 ^c^	4.25 ^b^	0.34	−0.345 *
Lactose	4.91 ^b^	5.12 ^a^	5.03 ^a^	4.88 ^b^	0.19	−0.294 *
BHBA (mmol L^−1^)	0.638 ^c^	0.998 ^b^	1.424 ^a^	0.968 ^b^	0.026	0.375 *
GLU (mmol L)	2.70 ^a^	2.37 ^b^	2.21 ^c^	2.36 ^b^	0.12	−0.345 *
LEP (ng mL^−1^)	2.57 ^c^	2.82 ^b^	2.92 ^a^	2.55 ^c^	0.09	−0.344 *

^a, b, c, d^—*p* ≤ 0.05; * *p* ≤ 0.05; EB—energy balance; BHBA—β-hydroxybutyrate; SL—stage of lactation, DMP—daily milk production, GLU—glucose, LEP—leptin.

**Table 3:** Indicators of the course of lipolysis and levels of pro-inflammatory markers in stages of lactation.

Parameter	Stage of Lactation (SL)				SEM	Correlation DL x
	SL1	SL2	SL3	SL4		
BCS	2.61 ^a^	2.34 ^b^	1.97 ^c^	2.09 ^c^	0.12	0.361 *
BCS%	−4.75 ^c^	−10.36 ^b^	−15.67 ^a^	+6.49 ^c^	0.19	−0.553 *
NEFA: (mmol L^−1^)						
C16:0	23.21 ^c^	26.43 ^b^	28.24 ^a^	24.95 ^c^	0.23	−0.435 *
C18:0	11.35 ^a^	11.39 ^a^	11.42 ^a^	10.72 ^b^	0.07	−0.458 *
C18:1-t9	1.81 ^d^	3.16 ^b^	3.58 ^a^	2.36 ^c^	0.11	−0.451 *
C18:2	1.68 ^c^	1.92 ^b^	2.10 ^a^	1.89 ^b^	0.09	−0.432 *
IL-6 (ng L^−1^)	94.46 ^c^	105.04 ^b^	111.58 ^a^	108.89 ^b^	0.94	−0.257 *
PL (ng L^−1^)	2.04 ^c^	2.50 ^b^	2.79 ^a^	2.53 ^b^	0.05	−0.395 *

^a, b, c, d^—*p* ≤ 0.05; * *p* ≤ 0.05; NEFAs—non-esterified fatty acids; DL—day of lactation; BCS—body condition score; BCS%—rate of BCS changes; PL—plasminogen, IL-6—interleukin-6.

**Table 4:** Values of the curvilinear correlation coefficient between the analyzed production indicators and NEB markers.

Parameter	SL1—3 (DL)	SL4 (DL)	GLU	LEP	IL-6	EB
BCS%	0.521 *	−0.621 *	−0.341 *	0.395 *	0.473 *	0.762 *
DMP (kg)	0.447 *	−0.343 *	−0.826 *	0.534 *	0.518 *	0.643 *
NEFAs: (mmol L^−1^)						
C16:0	0.447 *	−0.328 *	−0.536 *	0.643 *	0.638 *	0.636 *
C18:0	0.628 *	−0.455 *	−0.327 *	0.387 *	0.386 *	0.263 *
C18:1-t9	0.521 *	−0.334 *	−0.533 *	0.432 *	0.431 *	0.519 *
C18:2	0.487 *	−0.434 *	−0.519 *	0.392 *	0.572 *	0.378 *
BHBA (mmol L^−1^)	0.411 *	−0.224 *	−0.648 *	0.524 *	0.535 *	0.493 *
Glucose (mmol L^−1^)	−0.422 *	0.358 *	−	−0.487 *	−0.414 *	−0.356 *
Leptin (ng mL^−1^)	0.527 *	−0.425 *	−0.589 *	−	0.447 *	0.397 *
IL-6 (ng L^−1^)	0.428 *	−0.331 *	−0.486 *	0.257 *	−	0.288 *
PL (ng L^−1^)	0.398 *	−0.253 *	−0.619 *	0.359 *	0.528	0,428 *

* *p* ≤ 0.05; EB—energy balance; BCS—body condition score, DL—days of lactation; NEFAs—non-esterified fatty acids, SL—stage of lactation, BHBA—β-hydroxybutyrate, GLU—glucose, LEP—leptin, PL—plasminogen, IL-6—interleukin-6.

## Data Availability

Available from the corresponding author. Because of the participant consent obtained as part of the recruitment process, it is not possible to make these data publicly available.

## Abbreviations

EB	energy balance
AT	adipose tissue
BCS	body condition of cows
BCS%	rate of loss of body condition
BHBA	β-hydroxybutyrate
DMI	dry matter intake
DMP	daily milk production
FA	fatty acids
GLU	glucose
HF	Holstein-Friesian cows
IL-6	interleukin 6
LEP	leptin
LSM	least squares method
NEB	negative energy balance
NEFAs	non-esterified fatty acids
PL	plasminogen
SEM	standard error of the mean
SL	stages of lactation
TMR	total mixed ration

## References

[1] Adewuyi A.A., Gruys E., van Eerdenburg F.J. (2005). Non esterified fatty acids (NEFA) in dairy cattle. A review. Vet. Q..

[2] Kasimanickam R.K., Kasimanickam V.R., Olsen J.R., Jeffress E.J., Moore D.A., Kastelic J.P. (2013). Associations among serum pro-and anti-inflammatory cytokines, metabolic mediators, body condition, and uterine disease in postpartum dairy cows. Reprod. Biol. Endocrinol..

[3] Breukink H.J., Wensing T. (1998). Pathophysiology of the liver in high yielding dairy cows and its consequences for health and production. Bov. Pract..

[4] Loor J.J., Everts R.E., Bionaz M., Dann H.M., Morin D.E., Oliveira R., Rodriguez-Zas S.L., Drackley J.K., Lewin H.A. (2007). Nutrition-induced ketosis alters metabolic and signaling gene networks in liver of periparturient dairy cows. Physiol. Genom..

[5] Gordon J.L., Leblanc S.J., Duffield T.F. (2013). Ketosis treatment in lactating dairy cattle. Vet. Clin. N. Am. Food Anim. Pract..

[6] Kuhla B., Metges C.C., Hammon H.M. (2016). Endogenous and dietary lipids influencing feed intake and energy metabolism of periparturient dairy cows. Domest. Anim. Endocrinol..

[7] Turk R., Juretić D., Geres D., Svetina A., Turk N., Flegar-Mestrić Z. (2008). Influence of oxidative stress and metabolic adaptation on PON1 activity and MDA level in transition dairy cows. Anim. Reprod. Sci..

[8] Keane C.J., Hanlon A.J., Roche J.F., Burton J.L., Mee J.F., O’Doherty J.V., Sweeney T. (2006). Short communication: A potential antiapoptotic phenotype in neutrophils of cows milked once daily in early lactation. J. Dairy Sci..

[9] Kanehisa M., Goto S., Hattori M., Aoki-Kinoshita K.F., Itoh M., Kawashima S., Katayama T., Araki M., Hirakawa M. (2006). From genomics to chemical genomics: New developments in KEGG. Nucleic Acids Res..

[10] van Knegsel A.T., de Vries Reilingh G., Meulenberg S., van den Brand H., Dijkstra J., Kemp B., Parmentier H.K. (2007). Natural antibodies related to energy balance in early lactation dairy cows. J. Dairy Sci..

[11] Kosteli A., Sugaru E., Haemmerle G., Martin J.F., Lei J., Zechner R., Ferrante A.W. (2010). Weight loss and lipolysis promote a dynamic immune response in murine adipose tissue. J. Clin. Investig..

[12] Fantuzzi G. (2005). Adipose tissue, adipokines, and inflammation. J. Allergy Clin. Immunol..

[13] Loskutoff D.J., van Mourik J.A., Erickson L.A., Lawrence D. (1983). Detection of an unusually stable fibrinolytic inhibitor produced by bovine endothelial cells. Proc. Natl. Acad. Sci. USA.

[14] Zorio E., Gilabert-Estellés J., España F., Ramón L.A., Cosín R., Estellés A. (2008). Fibrinolysis: The key to new pathogenetic mechanisms. Curr. Med. Chem..

[15] Pedersen B.K. (2013). Muscle as a secretory organ. Compr. Physiol..

[16] Wankhade P.R., Manimaran A., Kumaresan A., Jeyakumar S., Sejian V., Rajendran D., Bagath M., Sivaram M., Ramesha K.P., Varghese M.R. (2019). Active immune system and dry matter intake during the transition period are associated with postpartum fertility in lactating Zebu cows. Livest. Sci..

[17] Keragala C.B., Medcalf R.L. (2021). Plasminogen: An enigmatic zymogen. Blood.

[18] Chana-Muñoz A., Jendroszek A., Sønnichsen M., Wang T., Ploug M., Jensen J.K., Andreasen P.A., Bendixen C., Panitz F. (2019). Origin and diversification of the plasminogen activation system among chordates. BMC Evol. Biol..

[19] Barthel D., Schindler S., Zipfel P.F. (2012). Plasminogen is a complement inhibitor. J. Biol. Chem..

[20] Baker S.K., Strickland S. (2020). A critical role for plasminogen in inflammation. J. Exp. Med..

[21] Faty A., Ferré P., Commans S. (2012). The acute phase protein Serum Amyloid A induces lipolysis and inflammation in human adipocytes through distinct pathways. PLoS ONE.

[22] Rega G., Kaun C., Weiss T.W., Demyanets S., Zorn G., Kastl S.P., Steiner S., Seidinger D., Kopp C.W., Frey M. (2005). Inflammatory cytokines interleukin-6 and oncostatin m induce plasminogen activator inhibitor-1 in human adipose tissue. Circulation.

[23] Contreras G.A., Strieder-Barboza C., De Koster J. (2018). Symposium review: Modulating adipose tissue lipolysis and remodeling to improve immune function during the transition period and early lactation of dairy cows. J. Dairy Sci..

[24] Karis P., Jaakson H., Ling K., Bruckmaier R.M., Gross J.J., Pärn P., Kaart T., Ots M. (2020). Body condition and insulin resistance interactions with periparturient gene expression in adipose tissue and lipid metabolism in dairy cows. J. Dairy Sci..

[25] Sabzikar Z., Mohri M., Seifi H.A. (2023). Variations of some adipokines, pro-inflammatory cytokines, oxidative stress biomarkers, and energy characteristics during the transition period in dairy cows. Vet. Res. Forum Int. Q. J..

[26] Kruithof E.K. (2008). Regulation of plasminogen activator inhibitor type 1 gene expression by inflammatory mediators and statins. Thromb. Haemost..

[27] Loskutoff D.J., Samad F. (1998). The adipocyte and hemostatic balance in obesity: Studies of PAI-1. Arterioscler. Thromb. Vasc. Biol..

[28] Yudkin J.S., Stehouwer C.D., Emeis J.J., Coppack S.W. (1999). C-Reactive protein in healthy subjects: Associations with obesity, insulin resistance, and endothelial dysfunction: A potential role for cytokines originating from adipose tissue?. Arterioscler. Thromb. Vasc. Biol..

[29] Zachut M. (2015). Defining the Adipose Tissue Proteome of Dairy Cows to Reveal Biomarkers Related to Peripartum Insulin Resistance and Metabolic Status. J. Proteome Res..

[30] AOAC International (2007). Official Methods of Analysis of AOAC International.

[31] Strzetelski J.E.D. (2009). The Nutritional Value of French and National Feed fur Ruminants.

[32] Sjaunja L.O., Baevre L., Junkkarinen L., Pedersen J., Setälä J.A. Nordic proposal for an energy corrected milk (ECM) formula. Proceedings of the 7th Session International Committee for Recording and Productivity of Milk Animals.

[33] Thompson A., Taylor B. (2008). Guide for the Use of the International System of Units (SI).

[34] NRC (National Research Council) (2001). Nutrient Requirements of Dairy Cattle, 7th rev. ed..

[35] Wildman E.E., Jones G.M., Wagner P.E., Boman R., Troutt H.F., Lesch T.N. (1982). A Dairy Cow Body Condition Scoring System and Its Relationship to Selected Production Characteristics. J. Dairy Sci..

[36] Hara A., Radin N.S. (1978). Lipid extraction of tissues with a low-toxicity solvent. Anal. Biochem..

[37] Kessel S., Stroehl M., Meyer H.H.D., Hiss S., Sauerwein H., Schwarz F.J., Bruckmaier R.M. (2008). Individual variability in physiological adaptation to metabolic stress during early lactation in dairy cows kept under equal conditions. J. Anim. Sci..

[38] Drackley J., Overton T., Douglas G. (2001). Adaptations of Glucose and Long-Chain Fatty Acid Metabolism in Liver of Dairy Cows during the Periparturient Period. J. Dairy Sci..

[39] Schoenberg K.M., Overton T.R. (2011). Effects of plane of nutrition and 2,4-thiazolidinedione on insulin responses and adipose tissue gene expression in dairy cattle during late gestation. J. Dairy Sci..

[40] Bobe G., Young J.W., Beitz D.C. (2004). Invited review: Pathology, etiology, prevention, and treatment of fatty liver in dairy cows. J. Dairy Sci..

[41] Stefan N., Häring H.U., Hu F.B., Schulze M.B. (2013). Metabolically healthy obesity: Epidemiology, mechanisms, and clinical implications. Lancet Diabetes Endocrinol..

[42] Chapinal N., Carson M.E., LeBlanc S.J., Leslie K.E., Godden S., Capel M., Santos J.E., Overton M.W., Duffield T.F. (2012). The association of serum metabolites in the transition period with milk production and early-lactation reproductive performance. J. Dairy Sci..

[43] Greenfield R.B., Cecava M.J., Donkin S.S. (2000). Changes in mRNA expression for gluconeogenic enzymes in liver of dairy cattle during the transition to lactation. J. Dairy Sci..

[44] Weber C., Hametner C., Tuchscherer A., Losand B., Kanitz E., Otten W., Sauerwein H., Bruckmaier R.M., Becker F., Kanitz W. (2013). Hepatic gene expression involved in glucose and lipid metabolism in transition cows: Effects of fat mobilization during early lactation in relation to milk performance and metabolic changes. J. Dairy Sci..

[45] Aschenbach J.R., Kristensen N.B., Donkin S.S., Hammon H.M., Penner G.B. (2010). Gluconeogenesis in dairy cows: The secret of making sweet milk from sour dough. Int. Union Biochem. Mol. Biol. Life.

[46] Trayhurn P. (2013). Hypoxia and adipose tissue function and dysfunction in obesity. Physiol. Rev..

[47] De Koster J.D., Opsomer G. (2013). Insulin resistance in dairy cows. Vet. Clin. N. Am. Food Anim. Pract..

[48] Eckel R.H., Grundy S.M., Zimmet P.Z. (2005). The metabolic syndrome. Lancet.

[49] Çolakoğlu H.E., Polat İ.M., Vural M.R., Kuplulu S., Pekcan M., Yazlık M.O., Baklaci C.U. (2017). Associations between leptin, body condition score, and energy metabolites in Holstein primiparous and multiparous cows from 2 to 8 weeks postpartum. Rev. Médecine Vétérinaire.

[50] Roh S.G., Suzuki Y., Gotoh T., Tatsumi R., Katoh K. (2016). Physiological Roles of Adipokines, Hepatokines, and Myokines in Ruminants. Asian-Australas. J. Anim. Sci..

[51] Kabara E., Sordillo L.M., Holcombe S., Contreras G.A. (2014). Adiponectin links adipose tissue function and monocyte inflammatory responses during bovine metabolic stress. Comp. Immunol. Microbiol. Infect. Dis..

[52] Wang H., Ye J. (2015). Regulation of energy balance by inflammation: Common theme in physiology and pathology. Rev. Endocr. Metab. Disord..

[53] Ye J., McGuinness O.P. (2013). Inflammation during obesity is not all bad: Evidence from animal and human studies. Am. J. Physiol. Endocrinol. Metab..

[54] Wallenius V., Wallenius K., Ahrén B., Rudling M., Carlsten H., Dickson S.L., Ohlsson C., Jansson J.O. (2002). Interleukin-6-deficient mice develop mature-onset obesity. Nat. Med..

